# Mucosal-Associated Invariant T Cells in Regenerative Medicine

**DOI:** 10.3389/fimmu.2017.01711

**Published:** 2017-12-01

**Authors:** Hiroshi Wakao, Chie Sugimoto, Shinzo Kimura, Rika Wakao

**Affiliations:** ^1^International Epidemiology, Dokkyo Medical University, Mibu, Japan; ^2^Office of Regulatory Science, Pharmaceutical and Medical Device Agency (PMDA), Tokyo, Japan

**Keywords:** mucosal-associated invariant T cells, induced pluripotent stem cells, infectious diseases, human immunodeficiency virus, cancer, cell therapy, regenerative medicine, drug resistance

## Abstract

Although antibiotics to inhibit bacterial growth and small compounds to interfere with the productive life cycle of human immunodeficiency virus (HIV) have successfully been used to control HIV infection, the recent emergence of the drug-resistant bacteria and viruses poses a serious concern for worldwide public health. Despite intensive scrutiny in developing novel antibiotics and drugs to overcome these problems, there is a dilemma such that once novel antibiotics are launched in markets, sooner or later antibiotic-resistant strains emerge. Thus, it is imperative to develop novel methods to avoid this vicious circle. Here, we discuss the possibility of using induced pluripotent stem cell (iPSC)-derived, innate-like T cells to control infection and potential application of these cells for cancer treatment. Mucosal-associated invariant T (MAIT) cells belong to an emerging family of innate-like T cells that link innate immunity to adaptive immunity. MAIT cells exert effector functions without priming and clonal expansion like innate immune cells and relay the immune response to adaptive immune cells through production of relevant cytokines. With these characteristics, MAIT cells are implicated in a wide range of human diseases such as autoimmune, infectious, and metabolic diseases, and cancer. Circulating MAIT cells are often depleted by these diseases and often remain depleted even after appropriate remedy because MAIT cells are susceptible to activation-induced cell death and poor at proliferation *in vivo*, which threatens the integrity of the immune system. Because MAIT cells have a pivotal role in human immunity, supplementation of MAIT cells into immunocompromised patients suffering from severe depletion of these cells may help recapitulate or recover immunocompetence. The generation of MAIT cells from human iPSCs has made it possible to procure MAIT cells lost from disease. Such technology creates new avenues for cell therapy and regenerative medicine for difficult-to-cure infectious diseases and cancer and contributes to improvement of our welfare.

## Introduction and Background

### Characteristics of Mucosal-Associated Invariant T (MAIT) Cells

Mucosal-associated invariant T cells are an emerging member of innate-like T cells that link innate immunity to adaptive immunity. MAIT cells are characterized by their expression of the semi-invariant T cell receptor (TCR, in most cases TRAV1-2-TRAJ33 in both humans and mice, and in a few cases TRAV1-2-TRAJ12 or TRAV1-2-TRAJ20 in humans) (1–3) and for their development dependence on the major histocompatibility complex (MHC) class 1b molecule, MHC class I-related protein (MR1), which is monomorphic in nature (4, 5). Although conventional T cells recognize peptidic antigens loaded on MHC class I or II, the antigens for MAIT cells are bacteria-born small compounds, such as derivatives of riboflavin (vitamin B2) biosynthesis pathways and folic acid biosynthesis (6, 7), or intermediate adducts from non-enzymatic spontaneous interactions between the derivative of vitamin B2 and metabolites such as glyoxal or methylglyoxal (8). MAIT cells produce a plethora of cytokines, such as interferon gamma (IFN-γ), tumor necrosis factor alpha (TNF-α), interleukin 17A (IL-17A), and IL-2, upon stimulation (9–11). MAIT cells express IL-18 receptor α chain (IL-18Rα) and CD161, a C-type lectin, concomitant with retinoic acid receptor-related orphan receptor C (RORC), which is associated with IL-17 secretion and high-level expression of IL-18Rα and CD161 (12). MAIT cells also harbor promyelocytic leukemia zinc finger (PLZF) responsible for the expansion and effector differentiation of natural killer T (NKT) cells, another member of the innate-like T cells (see below) (13, 14), but expression of these transcription factors is not confined to MAIT cells (7). In healthy humans, MAIT cells are mostly CD8^+^ or CD4^−^CD8^−^ (double negative) with few CD4^+^. Importantly, MAIT cells have an “effector/memory” phenotype (i.e., CD45RA^−^CD45RO^+^CD95^high^CD62L^low^) (9). Furthermore, it is conceivable that MAIT cells preferentially migrate to the liver and intestine because MAIT cells express α_4_β_7_ integrin, CCR9, and high levels of CCR5, CXCR6, and CCR6 but not CCR7 (5, 9, 15). NKT cells are another member of innate-like T cells and have been an intensive target for clinical translation (16, 17). Importantly, NKT cells recognize glycolipids derived from bacteria (*Sphingomonas wittichii*) presented on the MHC class I-like monomorphic molecule CD1d as an antigen through the semi-invariant TCR (16, 18). Because NKT cells produce copious amounts of an array of cytokines, such as IFN-γ, IL-4, and IL-10, upon activation, they are thought to control immune cell differentiation, which is a prerequisite for efficient adaptive immunity (16). Enthusiasm for NKT cells as a therapeutic target has further strengthened through the discovery of antitumor effects of NKT cells, but the mechanism for this has remained elusive (19). Both NKT cells and MAIT cells are evolutionally conserved between mice and humans (7), but their frequency significantly differs (17).

### MAIT Cells Are Abundant in Humans but Rare in Mice

Since the discovery of MAIT cells in 1993, it was not until 2009 that we learned the phenotype and function of MAIT cells in humans. This was largely because of the development of a monoclonal antibody that recognizes human MAIT cells and of MR1 tetramers that identify MR1-restricted cells (6, 20, 21). MAIT cells occupy 1–10, 2–10, and 20–50% of T cells in human peripheral blood, intestine, and liver, respectively (9). In sharp contrast, NKT cells represent at most 0.1% of T cells in human peripheral blood, but the frequency varies widely among people and tissues (22). Importantly, in mice the situation is reversed. NKT cells represent 0.5–1.0, 0.5–1.0, and 20–30% of T cells in mouse thymus, spleen, and liver, respectively, but MAIT cells only occupy up to 0.05, 0.08, and 0.6% in the corresponding tissues (2, 23). The difference in innate-like T cell frequency raises a critical concern when modeling human diseases in mice.

## Mait Cells in Human Diseases

Recent studies have revealed that MAIT cells are implicated in many diseases, such as infectious, autoimmune, and metabolic diseases, and cancer, but mechanistic insight into how MAIT cells have protective or deleterious roles in each disease has been not been described.

### MAIT Cells in Infectious Diseases

#### MAIT Cells in Bacterial Infections

Mouse infection models and studies in human infections have demonstrated that MAIT cells have a protective role. In a pulmonary infection model with the live vaccine strain *Francisella tularensis*, MAIT cells expand in the lung and produce the cytokines IFN-γ, TNF-α, and IL-17A, which are critical for controlling bacteria growth in an MR1-dependent manner. Importantly, MR1^−/−^ mice, which lack functional MAIT cells, have compromised bacterial clearance and delayed adaptive immune response in the lung but not in the spleen or liver (24). A similar increase in bacterial load has been observed in *Klebsiella pneumoniae* and *Mycobacteria bovis* BCG infection in MR1^−/−^ mice compared with that of wild-type mice (25, 26). An earlier study demonstrated the importance of MAIT cells in *Mycobacteria tuberculosis* infection in humans, and *Mycobacteria abscessus* and *Escherichia coli* infection using MAIT cell-specific TCR transgenic mice and in combination with MR1^−/−^ mice. Importantly, MAIT cells are depleted from peripheral blood and accumulate in the *M. tuberculosis-*infected lung of patients (21). Similar depletion of MAIT cells has been reported in individuals with active tuberculosis but not with latent infection, indicating that MAIT cells migrate to the infected lung only in the active disease (27). In addition, the remaining MAIT cells in the peripheral blood from active tuberculosis patients have compromised production of cytokines, such as IFN-γ and TNF-α, concomitant with higher expression of programmed death 1 (PD1), which indicates that *M. tuberculosis* infection not only depletes circulating MAIT cells but also undermines the effector function of MAIT cells (28). Furthermore, depletion of MAIT cells from peripheral blood is a risk factor in severely sick patients with sepsis for subsequent nosocomial infections and is correlated with the severity of cystic fibrosis, in particular, for those with chronic *Pseudomonas aeruginosa* infections (29, 30). These studies imply that MAIT cells somehow detect infection and migrate to the infection site where they may have a protective role. Given that the antigens for MAIT cells are compounds derived from bacteria-born vitamin B2 biosynthesis intermediates or adducts, it is not surprising that MAIT cells can detect bacterial infection in an MR1-dependent manner. However, MAIT cells can be activated by bacteria lacking the vitamin B2 biosynthesis pathway, such as in *Enterococcus faecalis* and *Streptococcus pyogenes*, probably in a TCR-independent manner (11). MR1-independent activation may be explained by stimulation of MAIT cells through innate pathways, such as IL-12 and IL-18, produced by monocytes or macrophages. This TCR-independent activation of MAIT cells and concomitant production of innate cytokines, IL-12 and IL-18, is important for protection against viral infections (see below).

#### MAIT Cells in Viral Infections

Mucosal-associated invariant T cells are not activated by viruses, such as encephalomyocarditis virus, Sendai virus, Newcastle disease virus, herpes simplex virus, and parainfluenza 3 virus, even though these viruses can activate dendritic cells (21). However, many reports have shown that the frequency of MAIT cells is significantly decreased during human viral infections such as human immunodeficiency virus (HIV) infections (31–36) and HIV/*M. tuberculosis* co-infections (37). Furthermore, MAIT cell frequency poorly recovers in peripheral blood despite successful combined antiretroviral therapy (cART), whereas rectal and colon CD8^+^ MAIT cells are relatively well conserved (31, 32). By contrast, CD4^+^ MAIT cells are lost in rectal mucosa concomitant with depletion of CD4^+^ T cells in HIV patients (32). Although the exact mechanism of MAIT cell depletion from peripheral blood is poorly understood, depletion may be caused by activation-induced cell death (AICD) of MAIT cells (31) or exhaustion and downregulation of CD161 (35). While the latter possibility may be aided by MR1-tetramer, CD8^+^ MAIT cells tend not to be vulnerable to HIV infection (35). The reason for MAIT cell depletion during HIV infection remains elusive and warrants further study. Because T helper type 17 (Th17) cells are depleted in simian immunodeficiency virus-infected rhesus macaques with concomitant defects in mucosal barrier function (38), it is likely that HIV patients with MAIT cell depletion will have compromised immune response against bacteria or virus, and eventually succumb to opportunistic infection. Depletion of MAIT cells from circulation has also been observed in influenza virus-infected and hepatic C virus-infected patients (39, 40). Intriguingly, in both cases, MAIT cells exhibit an activated phenotype in patients, indicating that MAIT cells play a protective role in combatting virus infections. However, because MAIT cells do not recognize virus-born peptides and RNA/DNA, this phenotype probably reflects TCR-independent activation. Indeed, MAIT cells are activated by IL-18 in synergy with IL-12, IL-15, and IFN-α/β in virus infections (39). Thus, MAIT cells have a critical role in host protection against bacteria and virus infections and serve as a target for clinical intervention for development of vaccines and adjuvants that bolster host immunity.

### MAIT Cells in Autoimmune Diseases

Similar to infections, the frequency of MAIT cells is often lower in patients with autoimmune diseases, such as multiple sclerosis (MS), inflammatory bowel disease (IBD), and entropathies, but the role of MAIT cells in these diseases remains elusive.

#### MAIT Cells in MS

Multiple sclerosis is an autoimmune disease characterized by inflammatory demyelination, gliosis, and axonal loss in the central nervous system (CNS) (41). Although autoreactive Th1 and Th17 have been suspected to cause diseases in the CNS, such as MS, neuromyelitis optica, and acute disseminated encephalomyelitis (42), the real pathogenic mechanism of MS is unknown. MAIT cells are depleted from peripheral blood in MS patients, particularly during relapse relative to remission (43, 44), but there is no difference in MAIT cell frequency between inactive and active MS patients (45) or even an increase during disease duration (46). FTY720 (fingolimod, a first-in-class drug for MS) therapy increases the relative frequency of MAIT cells (47). FTY720 is an antagonist of sphingosine-1-phosphate receptor and inhibits the egress of naive and central memory T and B cells from the lymph node. Importantly, FTY720 administration results in lymphopenia, in particular, in naive and central memory T and B cells harboring the chemokine receptor CCR7 (48). Because MAIT cells have an effector/memory phenotype, the increase of MAIT cells is not surprising, but the relative frequency of MAIT cells declines after prolonged treatment (47). Although CD8^+^ MAIT cells were found in postmortem brain lesion samples from MS patients, MAIT cells are depleted from peripheral blood concomitant with an increase in IL-18 in the serum, which suggests that MAIT cells migrate into the CNS lesion in MS patients (44). However, the role of MAIT cells in MS pathophysiology remains obscure. In contrast to human cases, the experimental autoimmune encephalomyelitis (EAE) murine MS model suggests that MAIT cells have a protective role in the pathogenesis of EAE because adoptive transfer of NK1.1^+^ cells (containing MAIT cells) prepared from transgenic mice with the MAIT cell-specific invariant TCR mitigates disease severity (49). In support of the above data, MR1^−/−^ mice that lack functional MAIT cells have more severe EAE, which again suggests a protective role for MAIT cells in EAE. Although the authors attributed this protective effect to the production of the anti-inflammatory cytokine IL-10 from B cells in an MR1-independent manner and contingent upon inducible T cell co-stimulator (ICOS)-B7-related protein interaction (49), these data should be interpreted with caution. Since NK1.1^+^ cells potentially contain not only authentic MAIT cells but also other cell types, determination of the precise role of MAIT cells in EAE requires a more elaborate mouse model, such as one in a TCR-Cα^−/−^ background, that assures the absence of other T cells for adoptive transfer.

#### MAIT Cells in IBDs

Because MAIT cells are preferentially located in the gut lamina propria of humans and mice (50), and MAIT development is dependent on commensal flora in mice (5), it is reasonable to assume that MAIT cells are implicated in gastrointestinal diseases, particularly those involved in mucosal immunity. X-linked lymphoproliferative (XLP) syndrome was the first identified inherited immunodeficiency associated with NKT cell defects. XLP is characterized by high susceptibility to Epstein–Barr virus infection, hemophagocytic lymphohistiocytosis, and hypogammaglobulinemia (51). XLP-1 is characterized by the deficiency of the signaling lymphocyte activation molecule (SLAM)-associated protein, and XLP-2 is caused by mutation in the X-linked inhibitor of apoptosis (XIAP) gene, which causes a significant loss of NKT and MAIT cells due to enhanced apoptosis (51, 52). Intriguingly, one out of six XLP-2 patients develops severe colitis (51). Because XIAP deficiency is correlated with an elevated risk of mortality in patients with colitis and also with enhanced apoptosis in MAIT cells, it suggests that MAIT cells have a protective role in colitis. Recent studies have demonstrated accumulation of MAIT cells in a lesion compared with that in a normal region within the ileum from patients with Crohn’s disease and in colons from active patients relative to non-active patients with ulcerative colitis (53, 54). In both cases, the frequency of circulating MAIT cells was lower in patients compared with that of healthy controls. Circulating MAIT cells are activated in colitis as evidenced by enhanced expression of Ki67, natural killer (NK) G2D, and B and T cell attenuator (BTLA) concomitant with increased IL-17 and IL-22 production (53) or by enhanced expression of CD69, IL-17, and IL-18 in the serum (54). Similar to MS, ligands that MAIT cells recognize in IBDs remain to be clarified. Identification of these ligands may reveal the role of MAIT cells in these diseases and whether MAIT cells have protective or deleterious effects.

#### MAIT Cells in Alloreactive Immune Responses

Mucosal-associated invariant T cells are also implicated in regulating immune response in allogeneic transplantation. MAIT cell number failed to recover after allogeneic hematopoietic cell transplantation in both myeloablative and non-myeloablative recipients. It appeared that inflammatory cytokines and gut microbacteria such as *Blautiaspp*. were required to promote MAIT cell reconstitution (55). MAIT cells also suppressed the proliferation of conventional T cells, indicating that MAIT cells could influence Graft-versus-host disease (GVHD) (55). On the contrary, a study on autologous hematopoietic stem cell transplantation has shown that MAIT cells were resistant to myeloablative conditioning. More than 30% patients have recovered MAIT cell number after transplantation by day 60. The more MAIT cells pretreatment patients possess, the less severe infectious complications (56). Thus, insight into the functions of MAIT cells in allogeneic transplantation would shed much light on the mechanisms of GVHD and open a novel horizon to suppress or to mitigate GVHD.

### MAIT Cells in Metabolic Diseases

Mucosal-associated invariant T cells have a pivotal role in obesity and type II diabetes mellitus (DM). Obesity affects the circulating and adipose tissue-resident immune system (57–61). Consequently, obesity causes sterile-inflammation, which is the cornerstone of the many diseases such as type II DM. NKT cells have an immunoregulatory role in adipose tissue in humans and mice (59). MAIT cells are also associated with obesity and type II DM in humans (62, 63). MAIT cells are depleted from the peripheral blood and enriched in adipose tissue in obese subjects, concomitant with enhanced IL-17 production. Although adipose-resident MAIT cells in non-obese subjects produce IL-10, an anti-inflammatory cytokine, the production is compromised in obese patients. Furthermore, MAIT cells in obese patients have an activated phenotype as evidenced by upregulation of CD25. Importantly, there is a positive correlation between insulin resistance and the frequency of MAIT cells, and MAIT cell frequency in peripheral blood recovers after bariatric surgery in obese patients (63). The above results suggest an inflammatory nature of MAIT cells in obesity and its strong connection to insulin resistance. Because intestinal transfer of microbiota from lean mice into the recipients with metabolic disease improves peripheral insulin sensitivity (64), it is thought that changes in microbiota in recipients alters MAIT cell function in gastrointestinal immune systems, which may also be relevant to IBDs.

### MAIT Cells in Cancer

Mucosal-associated invariant T cells have been implicated in kidney and brain tumors, and *TRAV1-2-TRAJ33* transcript is enriched in tumors relative to normal tissue and peripheral blood (65). Activated MAIT cells accumulate in adenocarcinoma and colorectal cancer with a compromised number of IFN-γ producing cells (66, 67). Although the degree of MAIT cell infiltration in the tumor inversely correlates with the life expectancy in adenocarcinoma patients (68), the frequency of circulating MAIT cells recovers after chemotherapy in patients with colorectal cancer (67). Similarly, after depletion of MAIT cells from peripheral blood in patients with mucosal-associated cancers, such as gastric, colon, and lung cancers, production of IFN-γ, IL-17, and TNF-α is maintained (69). MAIT cells have antiproliferative effects in a cancer cell line in a cell–cell contact-dependent manner and have cytolytic activity against the cancer cell line most likely *via* degranulation of granzyme B and perforin (67, 69). Nonetheless, how MAIT cells distinguish tumor cells from normal cells, and the molecular mechanisms through which MAIT cells exert antiproliferative and cytolytic activity has yet to be delineated.

## MAIT Cells Derived from Induced Pluripotent Stem Cells (iPSCs) for Cell Therapy and Regenerative Medicine

Although the function of MAIT cells in various diseases is beginning to emerge, targeting MAIT cells for clinical intervention remains challenging. The advent of iPSCs has made it possible to prepare an unlimited number of the desired cells or tissues for regenerative medicine in theory. Because MAIT cells are depleted in many diseases, such as infectious, autoimmune, and metabolic diseases, and cancer, it is plausible that depletion can be compensated by MAIT cells prepared from iPSCs or embryonic stem cells (ESCs). Furthermore, because MAIT cells are prone to AICD (31, 52), loss of MAIT cells under certain disease conditions will not recover even after recovery from disease. This hypothesis is further underpinned by the fact that the frequency of MAIT cells declines with age (70, 71), MAIT cells typically expand only 8–10 years after birth (9), which suggests a poor proliferative nature *in vivo* in adults.

### Generation and Phenotype of MAIT Cells from iPSCs (reMAIT Cells)

**Figure 1:**
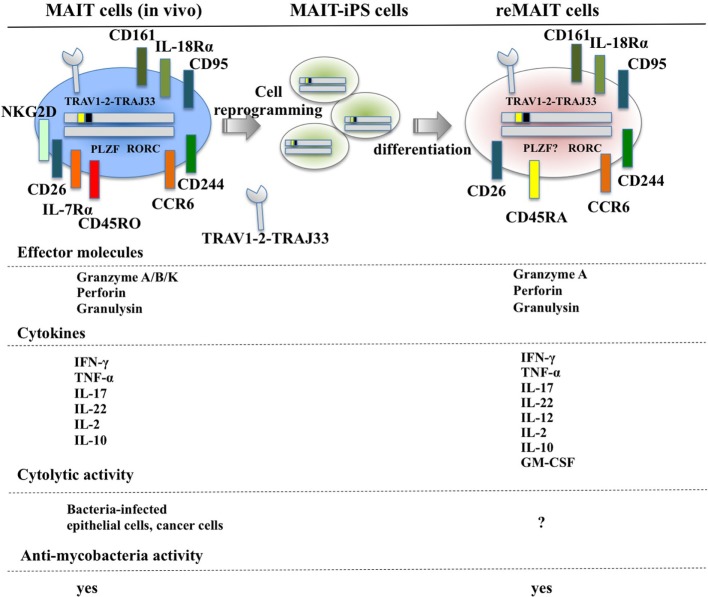
Cell surface antigens, effector molecules, cytokines, and effector functions of MAIT and reMAIT cells. reMAIT cell differentiation from MAIT cell-derived iPS cells (MAIT-iPS cells) *via* cell reprogramming of MAIT cells is illustrated (upper panel). Representative antigens and effector molecules present in MAIT cells (left panel) and in reMAIT cells (right panel) are shown. The transcription factors rich in MAIT cells, such as promyelocytic leukemia zinc finger (PLZF) and related orphan receptor C (RORC), are also depicted, but the presence of PLZF in reMAIT cells has not been determined. Effector molecules and cytokines from MAIT and reMAIT cells are shown. In addition, the target cells for the cytolytic activity and antimycobacterial activity of MAIT cells and reMAIT cells are indicated. “?” indicates not determined.

Although T cells can be differentiated from iPSCs or ESCs, they are not monoclonal in nature, which makes it difficult to directly apply this technology to cell therapy and regenerative medicine (72–75). In marked contrast, iPSCs prepared from human MAIT cells (MAIT-iPSCs) can generate MAIT-like cells under T cell-permissive culture conditions without ectopic gene expression (hereafter called reMAIT cells) (76). There is quasi-exclusive generation of reMAIT cells from MAIT-iPSCs, which indicates that configuration of the rearranged invariant TCRα chain locus specific for MAIT cells (TRAV1-2-TRAJ33) has a pivotal role in reMAIT cell fate determination during differentiation. Differentiation of reMAIT cells from MAIT-iPSCs does not accompany expression of the genes relevant to TCR rearrangement such as recombination-activating genes 1 and 2 and DNA nucleotidylexotransferase (77). Absence of these genes will inhibit further rearrangement of MAIT cell-specific TCR, which, in turn, preserves the identity of MAIT cells. Intriguingly, expression is silenced at both the epigenetic and transcriptional levels, further highlighting the role of rearranged TRAV1-2-TRAJ33 (77). reMAIT cells resemble MAIT cells in peripheral blood in that they express the invariant TCR (TRAV1-2-TRAJ33), CD161^high^, IL-18Rα^high^, CCR6^high^, CXCR3^+^, CXCR5^+^, and CD95 (Fas)^high^ with little expression of CCR7 (Figure 1). reMAIT cells harbor SLAM family proteins, such as CD150 and CD244 (78) together with CD26, and the dipeptidase is responsible for processing some chemokines and peptidic mediators (79–82). Although MAIT cells in peripheral blood from healthy donors have an effector/memory phenotype, reMAIT cells do not. In particular, they are CD127^low^ (IL-7Rα) and CD45RA^high^, which indicates that reMAIT cells are rather naive (Figure 1). Intriguingly, adoptive transfer of reMAIT cells into immunocompromised *NOD/Shi-scidIL-2R*γ*^−^*^/−^ (NOG) mice results in upregulation of certain chemokine receptors, such as CCR5, CXCR4, and CXCR6, relevant to tissue-specific migration (76). Furthermore, reMAIT cells represent the Th17 phenotype as evidenced by expression of CCR6, IL-12Rβ2, and IL-23R upon adoptive transfer (76, 83). Because reMAIT cells express CD161 and RORC and produce IL-17A before adoptive transfer, the above data strongly indicate that reMAIT cells have acquired Th17 nature during *in vitro* differentiation, but remain mostly double negative (CD4^−^CD8^−^) upon adoptive transfer (76).

### Functional Characterization of reMAIT Cells

Mucosal-associated invariant T cells from peripheral blood are activated by bacteria-infected monocytes and produce IFN-γ (21). Similarly, reMAIT cells are also activated upon anti-CD3/CD28 or PMA/ionomycin and produce a copious amount of IFN-γ upon coculture with bacteria-fed monocytes concomitant with expression of an array of cytokines and chemokines such as IL-10, TNF-α, IL-12p70, GM-CSF, IP-10, and MIG (76) (Figure 1). This cytokine and chemokine production profile suggests that reMAIT cells have a protective role in host defense against bacterial infection. In fact, adoptive transfer of reMAIT cells into NOG mice followed by *M. abscessus* inoculation inhibits bacterial growth in the liver and spleen, which demonstrates that reMAIT cells exert antibacterial activity *in vivo* (76). Further analysis has revealed that the human-specific effector granulysin is released in the serum upon infection (76). Granulysin has a pivotal role in antibacterial activity, in particular, through the delivery of granzymes into intracellular bacteria, and the subsequent cleavage of electron transport chain I and oxidative stress defense proteins leading to bacteria death (84). Because reMAIT cells harbor granzyme A, perforin, and granulysin, which are effector molecules required for cytolytic activity, reMAIT cells probably can kill bacteria.

### Potential Application of reMAIT Cells

Because reMAIT cells have antibacterial activity *in vivo*, these cells could be used for cell therapy against intractable infectious diseases. Furthermore, because MAIT cells exert anticancer activity, reMAIT hold great promise to treat cancer if anticancer activity is retained by reMAIT cells *in vivo*. In addition, reMAIT cells may be used to understand the underlying molecular mechanisms of MAIT cells in cancer. In the following section, we will discuss two potential possibilities for reMAIT cells in cell therapy.

#### reMAIT Cells for Severe Infection and HIV

**Figure 2:**
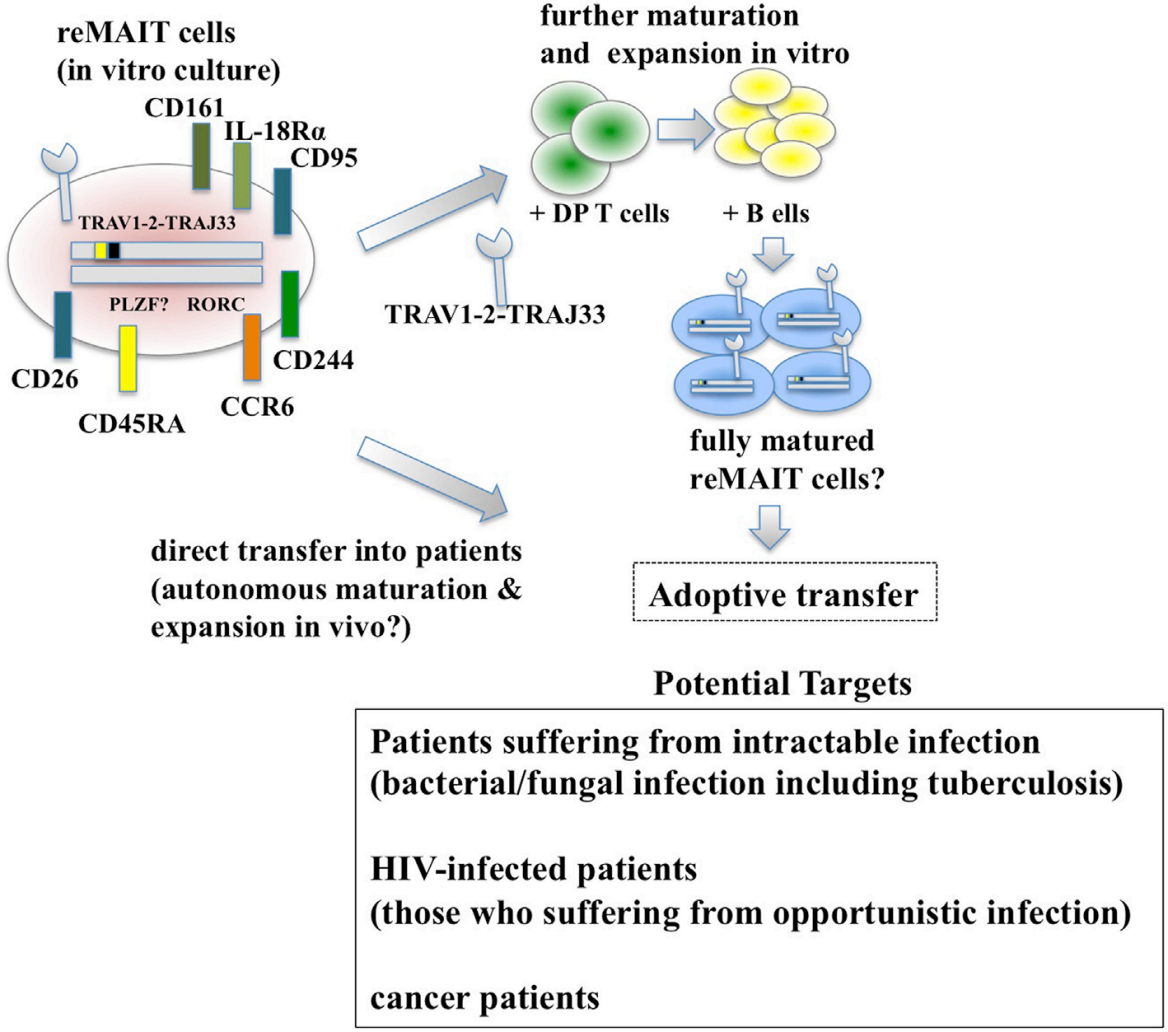
Adoptive transfer of reMAIT cells for cell therapy. Fully matured reMAIT cells could be prepared before adoptive transfer (upper panel) or directly transferred into the patient (middle panel). *In vitro*-cultured reMAIT cells could further be maturated and expanded through interaction with double-positive (DP) T cells followed by incubation with B cells *in vitro* before adoptive transfer (upper panel). Alternatively, reMAIT cells could directly be transferred into patients for autonomous maturation and expansion *in vivo* (middle panel). Potential targets for cell therapy with reMAIT cells are indicated (lower panel).

Historically, antibiotics have been the primary choice for the treatment of bacterial infections. However, the recent emergence of antibiotic-resistant bacteria poses a serious concern for global public health (85). Although much effort has been devoted to developing novel antibiotics, adjuvants that potentiate the immune system, antivirulent reagents that impair the establishment of bacterial infection, and biological therapies, such as monoclonal antibodies and agonists for innate immunity and the microbiome, more time and money is needed for these strategies to bear fruit. Because MAIT and reMAIT cells are protective in bacterial infections and MAIT cells exert cytolytic activity against bacterial-infected epithelial cells (86), reMAIT cells may be used to fight antibiotic-resistant bacterial infections and candidiasis where MAIT cell ligands are present. Given that patients who do not recover from severe sepsis tend to acquire nosocomial infections (29), supplementation of reMAIT cells into these patients may reinforce the immune system and mitigate the disease (Figure 2). The mechanism through which MAIT cells protect against infection consists of direct activation of MAIT cells *via* TCR followed by production of cytokines and chemokines that directly or indirectly exert antibacterial activity, activation *via* IL-12R and IL-18R in MAIT cells through the production of IL-12 and IL-18 from monocytes and macrophages, or some combination thereof (11, 26). TCR-dependent or TCR-independent activation of MAIT cells is important for recovering the loss of MAIT cells in HIV-infected subjects. Although the frequency and function of circulating MAIT cells is less in HIV-infected patients [reviewed in Ref. (87)], there is no decisive way to overcome these defects. Because reMAIT cells produce an array of cytokines and chemokines to fight bacterial infection and are competent to lyse epithelial cells infected with bacteria, cell therapy with reMAIT cells may further strengthen the efficacy of cART by suppressing opportunistic infection by *M. tuberculosis* or non-tuberculous mycobacteria such as *Mycobacterium avium* complex (MAC) (88). Even though cART results in a partial recovery of the cytokine production potential of MAIT cells upon *E. coli* stimulation, in particular IFN-γ and IL-17, TNF-α production remains perturbed (32). Furthermore, the number of circulating MAIT cells does not recover to that in healthy patients, even after successful cART, whereas rectal MAIT cells numbers seem to recover (34). A similar approach is possible to treat patients infected with MAC, for which no efficacious treatment has been available to date. Because the cause and underlying mechanism remain elusive, it is tempting to assume that reMAIT cell infusion reshapes an undermined immune system in HIV-infected (and mycobacteria-co-infected) patients in combination with cART.

#### reMAIT Cells for Cancer

Bacterial infection has a Janus-like feature—in most cases infection deteriorates health and threatens life, but in some select cases, intentional inoculation of bacteria, such as *Streptococcus erysipelatis*, a causative agent for the erysipelas, results in regression or complete disappearance of sarcoma (89). These seemingly contradictory results are important in that more detailed knowledge of bacterial infection and resultant immune reactions will aid understanding our immune system and open new avenues for cancer treatment (Figure 2). Although MAIT cells have been implicated in cancer, their precise role largely remains obscure. However, recent studies suggest that MAIT cells exert antiproliferative and even cytolytic activity against cancer cells in a cell–cell contact-dependent manner (67, 69). Because cytolytic activity of MAIT cells against bacteria-infected cells is dependent on MR1 (86), it is important to identify ligands that MAIT cells recognize through the cognate TCR in cancer. This not only will serve as a novel anticancer drug but also will expand a new horizon in MAIT cell biology—how MAIT cells distinguish normal cell from cancer cells. In particular, the underlying mechanism for BCG instillation, the standard therapy for bladder cancer and especially for non-muscle invasive bladder cancer, is still unknown (90) but may involve MAIT cells. Because MAIT cells are found in the urine of patients with urinary tract infection (UTI), and murine MAIT cells are recruited to the bladder upon bacterial infection and contribute to bacterial clearance in UTI model (91), it is plausible that BCG instillation somehow induces recruitment and activation of MAIT cells in bladder cancer, in particular at the mucosa or submucosa, and that leads to destruction of cancer cells (86, 91). Thus, studying MAIT cells in bladder cancer will provide not only mechanistic insight into the role of BCG but also be pertinent to the disease state and efficacy of BCG in patients, which could serve as a biomarker for diagnosis or prognosis. Irrespective of a patient’s infection state, whether reMAIT infusion confers anticancer activity and inhibits tumor growth awaits future study. It is worth noting that reMAIT cells would have to be considered for specific cancers in which MAIT cell ligands are available, such as colorectal cancer or in combination with microbe-based therapy as discussed earlier, because the cytolytic activity of MAIT cells would be dependent on microbial ligands presented on MR1. The use of reMAIT cells in cancer treatment is still its infancy and warrants further study.

## The Premise for Cell Therapy

reMAIT cells hold great promise for cell therapy against bacterial and viral infections, and cancer, if reMAIT cells behave identically to authentic MAIT cells *in vivo* upon adoptive transfer. This premise and successful implementation with reMAIT cells depends on the following issues: first, MAIT-iPSCs and the resultant reMAIT cells should be free from genomic modification, in particular, from amplification of potential oncogenes and from deprivation of suppressor oncogenes. Second, undifferentiated cells and other type of cells generated during reMAIT differentiation should be carefully eliminated, as contamination with the former poses a risk of teratoma and that with latter compromises the efficacy and function of reMAIT cells. Third, reMAIT cells need to maintain their identity, and mimic or acquire proper function *in vivo*, including cell surface antigen expression profile, cytokine/chemokine production ability, cytolytic activity, and transcriptional and epigenetic state. Finally, reMAIT cells that persist in recipients (not rejected by the recipient’s immune system) should circulate in the blood and migrate to proper tissues. The first criterion can be monitored by RNA-seq with a help of next generation sequencing technology in MAIT-iPSCs and reMAIT cells [for example, Ref. (92)]. Purification of reMAIT cells can be achieved through fluorescent activated cell sorting or magnet-based cell purification procedures. Because reMAIT cells derived from MAIT-iPSCs harbor a phenotype quasi-identical to MAIT cells and produce cytokines and chemokines as MAIT cells upon adoptive transfer, it is conceivable that reMAIT cells are ready to be used in cell therapy. This view is further underpinned by the fact that reMAIT cells are devoid of machinery responsible for TCR rearrangement such as recombination-activating genes (77). The last criterion can be met through the use of autologous MAIT cells for MAIT-iPSC preparation and supported by adoptive transfer of reMAIT cells into immunocompromised mice to observe maturation of reMAIT cells and cytokine and chemokine production *in vivo*. It has already been shown that reMAIT cells can migrate to various tissues in immunocompromised mice (76), which suggests functional activity. The above data are promising for implementation of cell therapy with reMAIT cells, but some caveats remain.

## Caveats in the Use of reMAIT Cells for Therapy and Regenerative Medicine

**Figure 3:**
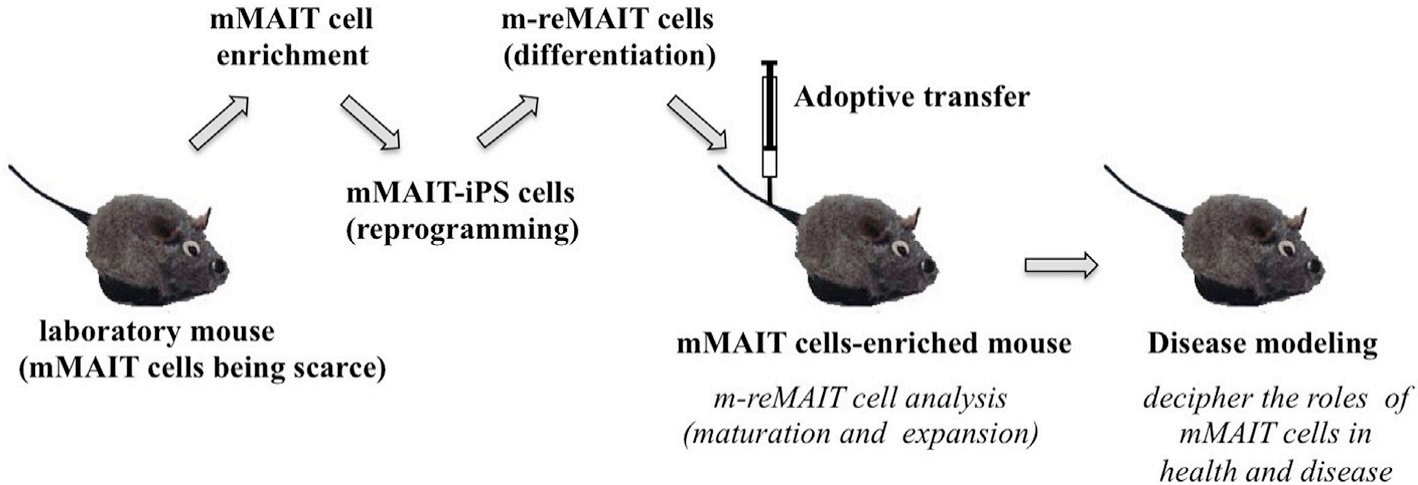
A novel mouse model for studying the role of MAIT cells in health and disease. The strategy to generate mice rich in MAIT cells is summarized. Mouse (m) MAIT cells are enriched from laboratory mice (e.g., C57BL/6) by combination of cell surface antigens. mMAIT-enriched fractions are subjected to cell reprogramming to generate iPS cells. Induced pluripotent stem cells from mMAIT cells (referred as mMAIT-iPS cells) are differentiated into mouse MAIT cells (referred as m-reMAIT cells) *via* a standard T cell lineage differentiation protocol. Maturation and expansion of m-reMAIT cells are assessed in congenic mice upon adoptive transfer. Once m-reMAIT cells settle in congenic mice, mice could be used to model various diseases, such as infectious, autoimmune, and metabolic diseases, and cancer, to decipher the role of mMAIT cells.

While reMAIT cells autonomously mature to a certain degree in the immunocompromised mice, there is little expression of molecules relevant for activation, such as CD127 (IL-7R), and for costimulation such as CD27 and CD28. Furthermore, the expression profile of CD150, CD244, CD26, NKG2D, and CD95 in reMAIT cells in these mice indicates that they are not identical to MAIT cells present in peripheral blood (9, 76). This difference most likely reflects the absence of immune cells in immunocompromised mice. In fact, MAIT cells require double-positive thymocytes for positive selection in the thymus, and B cells for peripheral expansion and maturation (5, 93). Thus, it is imperative to let reMAIT cells interact with these cells *in vitro* or *in vivo* for successful reconstitution of MAIT cell function (Figure 2). Currently, there is no animal model for studying the functional maturation and expansion of reMAIT cells. Therefore, generation of iPSCs from mouse MAIT cells is an alternative to overcome the above dilemma (Figure 3). Although MAIT cells are scarce in mouse, the derived iPSCs are invaluable for generating murine reMAIT cells for adoptive transfer. It is reasonable to assume that the maturation and expansion of reMAIT cells can be analyzed in mice without immune rejection, provided that iPSCs and the resultant reMAIT cells (donor) are prepared from the same recipient mouse (congenic mice). This technology will provide a novel animal model in which an increased frequency of MAIT cells is guaranteed without using a transgene such as invariant TCR. Because the frequency of innate-like T cells, such as NKT and MAIT cells, differs significantly between humans and mice, the immune environment in laboratory mice does not mirror that in humans (17), which may result in a different physiological outputs. It is possible that the role of NKT cells is overemphasized, while that of MAIT cells is underappreciated, in current mice models. Thus, the advent of novel mice with abundant MAIT cells will not only facilitate deciphering the role of MAIT cells in health and disease but also make possible the development of pharmaceutical products targeting MAIT cells. This type of animal model will serve as a scaffold for implementing clinical trials in humans, and open up new avenues to treat unmet medical needs and diseases.

## Conclusion

Although engineered T cells, such as cytotoxic T cells with chimeric antigen receptor and antigen-specific cytotoxic T cells from iPSCs, have been used to treat cancer, MAIT cells are unique in that they are quite abundant in humans and that their antigens are small compounds, which suggests that targeting MAIT cells may be beneficial for developing new cancer treatments (17, 94–97). Furthermore, the intimate relationship between depletion of MAIT cells from circulation and migration to diseased sites suggests that MAIT cells are poor at proliferation *in vivo* and that procurement of reMAIT cells would alleviate diseases where MAIT cell numbers do not recover. Thus, reMAIT cells hold great promise to treat a myriad of diseases, and deciphering the role of MAIT cells in health and disease is needed.

## Author Contributions

HW, CS, SK, and RW contributed to the conception and draft of the work, approved the final version to be published, and agreed to be accountable for all aspects of the work in ensuring that questions related to the accuracy or integrity of any part of the work are appropriately addressed and interpreted.

## Conflict of Interest Statement

The authors declare that the research on reMAIT cells has been conducted in collaboration with Asubio Pharma Co. (Daiichi-Sankyo Co.). However, Asubio Pharma Co. had no role in study design, data collection, analysis, decision to publish, or preparation of manuscript.
